# Spatial Variation in Genetic Diversity and Natural Selection on the Thrombospondin-Related Adhesive Protein Locus of *Plasmodium vivax* (*PvTRAP*)

**DOI:** 10.1371/journal.pone.0110463

**Published:** 2014-10-21

**Authors:** Rattiporn Kosuwin, Chaturong Putaporntip, Hiroshi Tachibana, Somchai Jongwutiwes

**Affiliations:** 1 Molecular Biology of Malaria and Opportunistic Parasites Research Unit, Department of Parasitology, Faculty of Medicine, Chulalongkorn University, Bangkok, Thailand; 2 Department of Infectious Diseases, Tokai University School of Medicine, Kanagawa, Japan; Université Pierre et Marie Curie, France

## Abstract

Thrombospondin-related adhesive protein (TRAP) of malaria parasites is essential for sporozoite motility and invasions into mosquito’s salivary gland and vertebrate’s hepatocyte; thereby, it is a promising target for pre-erythrocytic vaccine. TRAP of *Plasmodium vivax* (PvTRAP) exhibits sequence heterogeneity among isolates, an issue relevant to vaccine development. To gain insights into variation in the complete *PvTRAP* sequences of parasites in Thailand, 114 vivax malaria patients were recruited in 2006–2007 from 4 major endemic provinces bordering Myanmar (Tak in the northwest, n = 30 and Prachuap Khirikhan in the southwest, n = 25), Cambodia (Chanthaburi in the east, n = 29) and Malaysia (Yala and Narathiwat in the south, n = 30). In total, 26 amino acid substitutions were detected and 9 of which were novel, resulting in 44 distinct haplotypes. Haplotype and nucleotide diversities were lowest in southern *P. vivax* population while higher levels of diversities were observed in other populations. Evidences of positive selection on *PvTRAP* were demonstrated in domains II and IV and purifying selection in domains I, II and VI. Genetic differentiation was significant between each population except that between populations bordering Myanmar where transmigration was common. Regression analysis of pairwise linearized *Fst* and geographic distance suggests that *P. vivax* populations in Thailand have been isolated by distance. Sequence diversity of *PvTRAP* seems to be temporally stable over one decade in Tak province based on comparison of isolates collected in 1996 (n = 36) and 2006–2007. Besides natural selection, evidences of intragenic recombination have been supported in this study that could maintain and further generate diversity in this locus. It remains to be investigated whether amino acid substitutions in PvTRAP could influence host immune responses although several predicted variant T cell epitopes drastically altered the epitope scores. Knowledge on geographic diversity in PvTRAP constitutes an important basis for vaccine design provided that vaccination largely confers variant-specific immunity.

## Introduction

In low- and middle-income countries in tropical areas, malaria remains one of the leading ten causes of morbidity and mortality, resulting in an estimated economic loss of nearly 40 million disability-adjusted life-years (DALYs) [1]. Although *Plasmodium falciparum* is the most pernicious and prevalent species, the significance of *P. vivax* should not be underappreciated because it can cause chronic relapsing illness due to reactivation of hypnozoites and it can potentially lead to severe complications similar to those caused by *P. falciparum* [2]. Furthermore, vivax malaria occupies a broader geographical range than falciparum malaria whilst the emergences of chloroquine- and primaquine-resistant *P. vivax* are of particular concern if they would be wide-spreading as chloroquine-resistant *P. falciparum* [3]. The progress of malaria control and elimination has been hampered by not only the emergence of drug resistant parasites but also insecticide resistant mosquito vectors; thereby, development of a malaria vaccine offers an important preventive measure [4]. Because *P. vivax* and *P. falciparum* co-circulate in several endemic areas outside Africa where co-infections of both species are not uncommon [5], effective malaria control requires vaccines against both species.

To date, malarial circumsporozoite (CS) protein is a prime candidate for pre-erythrocytic vaccine development [4]. Although CSP-derived immunogens could elicit immunity against sporozoites, the subunit vaccines derived from this molecule such as RTS, S recombinant vaccine against *P. falciparum* has resulted in limited clinical efficacy in field studies [6]. Because vaccines derived from irradiation-attenuated or live sporozoites consistently outperform vaccines incorporating single sporozoite proteins, a more effective pre-erythrocytic stage vaccine may require combination of multiple protective immunogens [7], [8]. In a murine model, co-immunization of CSP with thrombospondin-related adhesive protein (TRAP), a protein mobilized from microneme to the surface of sporozoite [9], has conferred complete protection against parasite challenge whereas vaccination using each of these immunogens could elicit only partial protection [10]. It is important to note that TRAP-specific CD8+ T lymphocytes are prime mediators for protection against sporozoite challenge in mouse vaccination trials, resulting in significant reduction in liver stage parasites [11]. Furthermore, seroepidemiological study has shown that anti-TRAP antibodies were negatively correlated with parasite density among infected individuals in malaria endemic areas [12].

TRAP has been shown to mediate gliding motility and invasion processes of malarial sporozoites into vertebrate’s hepatocyte and mosquito’s salivary gland [13], [14]. TRAP contains a hydrophobic N-terminal peptide (domain I), an integrin-like magnesium binding (or von Willebrand factor) A domain (domain II), thrombospondin type I repeats (domain III), an acidic proline/asparagine-rich region (domain IV), hydrophobic transmembrane domain (domain V) and a cytoplasmic tail (domain VI) [15], [16]. The locomotion of sporozoites is mediated by the subpellicular actomyosin system that linked to the cytoplasmic tail of TRAP [17]. Despite functional importance of TRAP in parasite survival, analysis of the TRAP loci of *P. falciparum* (PfTRAP) and of *P. vivax* (PvTRAP) from clinical isolates revealed microheterogeneity of sequence that has been maintained by positive selective pressure [18]–[20]. Although it remains to be explored whether polymorphism in T cell epitopes of malarial TRAP could alter host cell immune recognition as that observed in CSP of *P. falciparum* [21], a rationale design of a malaria subunit vaccine targeting TRAP requires knowledge on the extent and pattern of sequence diversity in this molecule from diverse endemic areas.

Both *P. vivax* and *P. falciparum* have been circulating in Thailand with almost comparable prevalence since the past 2 decades. However, regional prevalence of *P. vivax* relative to *P. falciparum* seems to differ across major endemic areas of Thailand [22], [23]. Our recent studies have shown that *P. vivax* populations in this country exhibited spatial variation in the extent of sequence diversity of major malaria vaccine candidate loci [24], [25]. An effective malaria vaccine design undoubtedly requires knowledge on this issue to circumvent possible vaccine escape variants. Herein, we analyzed sequence diversity in the PvTRAP locus of clinical isolates from 4 major malaria endemic areas of Thailand.

## Materials and Methods

### Human Ethics Statement

The study protocol was approved by the Institutional Review Board on Human Research of Faculty of Medicine, Chulalongkorn University (IRB303/56). Written informed consent was obtained from all participants.

### Parasite populations

Blood samples were obtained from 114 *P. vivax*-infected patients in northwestern (Tak province, n = 30), southwestern (Prachuap Khirikhan province, n = 25), eastern (Chanthaburi province, n = 29) and southern (Yala and Narathiwat provinces, n = 30) Thailand during 2006 and 2007. Venous blood samples were taken from each patient and were preserved in EDTA anticoagulant. These isolates have been previously determined to contain single clone infection based on sequence analysis of the merozoite surface protein-1 gene (*Pvmsp-1*) as described previously [24], [26].

### DNA extraction, amplification and sequencing

DNA was prepared from blood samples using QIAamp kit (Qiagen, Hilden, Germany). The complete *PvTRAP* sequence was amplified by nested PCR using a forward outer primer (PvTRAP-F0: 5′-ATGTGTGTACATTTGCGTATG-3′, nucleotides −442 to −422 before the start codon of the Salvador I sequence, GenBank accession number XM_001614097) and a reverse outer primer (PvTRAP-R0: 5′-TCATGAAGTGGCGAAACAAAC-3′, nucleotides 1961 to 1968 from the start codon). The inner pairs of primers were PvTRAP-F1 (5′-ATGTCTGTGTGAGCGCGCGGT-3′, nucleotides −398 to −378) and PvTRAP-R1 (5′-TCATCAGAAGCAGTTCCAAG-3′, nucleotides 1791 to 1810). PCR amplification was performed in a total volume of 30 µl of the reaction mixture containing template DNA, 2.5 mM MgCl_2_, 300 mM each deoxynucleoside triphosphate, 3 µl of 10x ExTaq PCR buffer, 0.3 µM of each primer and 1.25 units of ExTaq DNA polymerase (Takara, Seta, Japan). Thermal cycling profile for primary PCR included the preamplification denaturation at 94°C for 2 min followed by 35 cycles of 94°C for 30 s, 55°C for 30 s and 72°C for 2 min, and a final extension at 72°C for 5 min. Nested PCR was done using the same thermal profile except that amplification was done for 25 cycles. DNA amplification was carried out by using a GeneAmp 9700 PCR thermal cycler (Applied Biosystems, Foster City, CA). ExTaq DNA polymerase reportedly possesses efficient 5′→3′ exonuclease activity to increase fidelity and no strand displacement (Takara, Japan). The PCR product was examined by electrophoresis in a 1% agarose gel and was purified by using QIAquick PCR purification kit (QIAGEN, Germany). The PvTRAP sequences were determined directly and bi-directionally from PCR-purified templates and were performed on an ABI3100 Genetic Analyzer using the Big Dye Terminator v3.1 Cycle Sequencing Kit (Applied Biosystems, USA). Whenever singleton substitution occurred, sequence was re-determined using PCR products from two independent amplifications from the same DNA template. Sequences in this study have been deposited in the GenBank Database under the accession numbers KJ807186 to KJ807299.

### Statistical analyses

Sequence alignment with the Clustal X program [27] was done by using the *PvTRAP* sequence of the Salvador I strain as reference. The *PvTRAP* sequences of our previous Thai and Brazilian isolates previously published [19] and those reported by others [28] were included for analysis. For interspecific comparison, all *PvTRAP* sequences were aligned with the TRAP sequences of *P. knowlesi* (*PkTRAP*) and *P. cynomolgi* (*PcyTRAP*) (GenBank accession numbers XM_002259951 and XM_004223600, respectively). Haplotype diversity (*h*) and its sampling variance were computed according to equations 8.4 and 8.12 but replacing 2n by n [29]. Nucleotide diversity (π) was computed from the average number of pairwise sequence differences in the sample [29] using the MEGA version 6 program [30]. Evidence of genetic recombination was determined by 2 different approaches: (i) estimation of the minimum number of recombination events (Rm) that can be parsimoniously inferred from a sample of sequences with Monte Carlo simulations [31] using the DnaSP version 5 software [32] and (ii) searching for recombination breakpoints by phylogenetic approach by the Genetic Algorithm Recombination Detection (GARD) method. Goodness of fit for the GARD method was calculated by Akaike Information Criterion derived from a maximum likelihood model fit to each segment (AICc) using the HyPhy package [33], [34].

The rates of synonymous substitutions per synonymous site (*d*_S_) and nonsynonymous substitutions per nonsynonymous site (*d*_N_) were computed by using Nei and Gojobori’s method [35] with Juke and Cantor correction [36] and their standard errors of these parameters were estimated by the bootstrap method with 1,000 pseudoreplicates as implemented in the MEGA 6.0 program [30]. A statistical significance level of differences in *d*_S_ and *d*_N_ was set at 5%.

Tests for departure from neutral evolution based on intraspecific comparisons were done by Tajima’s *D*, Fu and Li’s D* and F* statistics. Tajima’s *D* test determines the differences between the average number of nucleotide differences and an estimate of θ from the number of segregating sites where θ = 4*Neµ*, in which *Ne* and µ are the effective population size and the mutation rate, respectively [37]. Fu and Li’s statistics measure the differences between the number of singletons and the total number of mutations (D* test) and the differences between the number of singletons and the average number of nucleotide differences between pairs of sequences (F* test) [38]. To examine whether deviation from neutrality occurs in particular fragments of *PvTRAP*, a sliding window analysis of Tajima’s *D* across the coding sequences was done using a window size of 250 bp and a step size of 10 bp [32].

Evidence of departure from neutrality based on interspecific comparsion included Fu and Li’s *D* and *F* tests, and McDonald-Kreitman tests. Fu and Li’s *D* statistics measure the differences between the number of mutations in external branches of the genealogy and the number of mutations while their *F* statistic compares the differences between the number of mutations in external branches of the genealogy and the average number of nucleotide differences between pairs of sequences [38]. Coalescence simulation with 10,000 pseudoreplicates was done to estimate significance levels of these parameters as implemented in the DnaSP program [32]. The McDonald-Kreitman test contrasts intraspecific polymorphism with interspecific divergence of closely related species. Statistical departure from neutral expectation was computed by Fischer’s exact test [39].

Tests of departure from neutrality at specific codons were performed based on estimation of the global ratio of the rate of non-synonymous to synonymous substitutions (dN/dS) across the *PvTRAP* gene. The single-likelihood ancestor counting (SLAC), fixed effects likelihood (FEL), random effects likelihood (REL) and mixed effects model of evolution (MEME) methods implemented in the HyPhy package [33] were used for analysis. SLAC model is highly conservative based on the maximum likelihood reconstruction of the ancestral sequences and the counts of synonymous and nonsynonymous changes at each codon position in a phylogeny under the assumption of neutral evolution. FEL model compares the ratio of nonsynonymous to synonymous substitution on a site-by-site basis, without assuming an *a priori* distribution of rates across sites whereas REL model first fits a distribution of rates across sites and then infers the substitution rate for individual sites. MEM algorithm detects codons under episodic positive selection unmasked by the abundance of purifying selection along the lineages [40]. Significance level settings for SLAC, FEL and MEME were *p* values<0.1 and Bayes Factor>1000 for REL followed the default values available on the Datamonkey Web Server [41].

The population genetic structure was analyzed by molecular variance approach (AMOVA) using the Arlequin 3.5 software which is similar to the Weir and Cockerham’s method but it takes into account the number of mutations between haplotypes [42]. The implemented algorithm calculates the fixation index *F*_ST_ identical to the weighted average *F*-statistic over loci, θw [43] and the significance levels of the fixation indices were estimated by a non-parametric permutation [42]. Pairwise Slakin’s linearized *Fst* was calculated based on *Fst*/(1-*Fst*) to address the relationship between the magnitude of population differentiation and distance between endemic areas [44].

The genetic structure was also determined by STRUCTURE 2.3.4 program that deploys the Bayesian model–based clustering approach [45]. The most probable number of populations (K) was estimated using an admixture model. All sample data were run for values K = 1–10, each with a total of 15 iterations. We used 500,000 Markov Chain Monte Carlo generations for each run following a burn-in of 50,000 steps. The most likely number K in the data was estimated by calculating ΔK values [46] and identifying the K value that maximizes the log probability of data, lnP(D) [45] as implemented in STRUCTURE HARVESTER program [47].

The phylogenetic tree of the *PvTRAP* haplotypes was constructed by using the Maximum Likelihood method based on the model with the lowest Bayesian Information Criterion (BIC) scores [30]. All positions containing gaps and missing data were eliminated. Initial tree(s) for the heuristic search were obtained by applying Neighbor-Join and BioNJ algorithms to a matrix of pairwise distances estimated using the Maximum Composite Likelihood (MCL) approach. The final tree topology was selected based on superior log likelihood value as implemented in the MEGA version 6 program [30].

Amino acid properties were characterized based on polarity and charge. Polarity of each residue is categorized as polar (S, Y, C, W, H, Q, T and N) or nonpolar (the remainder). Charge property includes positive (H, R and K), negative (D and E) or neutral (the remainder). Prediction of HLA-I binding peptides was based on the method taken into account proteasomal C terminal cleavage and transporter associated with antigen processing (TAP) transport efficiency. The predicted scores were determined by the NetCTL program [48].

## Results

### Genetic diversity in *PvTRAP*

Of 114 complete *PvTRAP* sequences, 109 isolates contained 1668 bp and 5 isolates from Tak province whose sequences were identical had 1677 bp. Sequence alignment with the Salvador I strain revealed 36 nucleotide substitutions within 33 codons, resulting in 26 nonsynonymous and 7 synonymous changes. Comparing with previous data from Brazilian, Thai [19], South Korean isolates [28], 9 new amino acid substitutions were identified (Table 1). Like our previous findings, the majority of amino acid changes occurred in domains II and IV [19]. Three codons contained more than two substituted nucleotides: codon 206 (after the Salvador 1 sequence) having 4 different substitutions: c.616C>T (P206S), c.617C>G (P206S), c.617C>T (P206L) and c.616_617delinsTT (P206L), codon 268 having double mutations: c.802_803delinsGG (K268G), and codon 424 containing 2 changes: c.1271A>C (N424T) and c.1272C>A (N424K). Among isolates examined herein, no discernible bias in frequency of nucleotide substitutions in each position of the codons occurred, i.e. 11, 11 and 13 substitutions in the first, second and third positions of the codons, respectively. In total, 44 distinct haplotypes were identified in this analysis, one of which had a duplication of 9 nucleotides encoding PDS in domain IV as previously noted [19] and responsible for size variation (Tables 1 and S1).

**Table 1:** Distribution of PvTRAP haplotypes from 4 major malaria endemic areas of Thailand.

	Amino acid residue																													Tak	PrachuapKhirikhan	Chanthaburi	Yala &Narathiwat	Total
			1	1	1	1	1	1	1	1	1	1	1	1	2	2	2	2	3	3	3	3	3	3	4	4	4	4	4					
Haplotype	1	4	2	2	3	3	3	4	6	6	7	7	8	8	0	5	6	9	2	3	4	8	8	8	1	2	4	4	6					
	5	5	0	2	4	5	7	3	0	6	2	6	0	6	6	5	8	7	4	6	0	3	4	5	3	4	2	3	7					
Domain	I	II	II	II	II	II	II	II	II	II	II	II	II	II	II	III	III	IV	IV	IV	IV	IV	IV	IV	IV	IV	IV	IV	IV					
Salvador-1	L	L	T	T	D	E	Q	R	V	R	N	Q	S	V	P	R	K	T	D	G	N	-	-	-	N	N	G	R	E					
#1	**.**	**.**	S	S	E	**.**	**.**	**.**	I	T	K	.	.	I	**.**	.	G	.	**.**	R	**.**	-	-	-	.	.	D	**.**	.	3	4	-	-	7
#2	**.**	**.**	S	S	.	**.**	**.**	**.**	I	T	K	.	.	I	**.**	K	G	.	**.**	R	**.**	-	-	-	.	.	D	**.**	.	3	-	-	-	3
#3	**.**	**.**	S	S	E	**.**	**.**	**.**	I	T	K	.	.	I	**.**	K	G	.	**.**	R	**.**	-	-	-	.	.	.	**G**	.	2	-	-	-	2
#4	**.**	**.**	S	S	E	**.**	**.**	**.**	I	T	K	.	.	I	**.**	.	G	.	**.**	R	**.**	-	-	-	K	T	.	**.**	A	1	-	-	-	1
#5	**.**	**.**	S	S	E	**.**	**.**	**.**	I	T	K	.	.	I	**.**	.	G	.	**.**	R	**.**	P	D	S	K	.	D	**.**	.	5	-	-	-	5
#6	**.**	**.**	S	S	.	**.**	**.**	**.**	.	.	K	.	.	I	**.**	.	G	.	**.**	R	**.**	-	-	-	.	.	.	**G**	.	3	1	-	-	4
#7	**.**	**.**	S	S	E	**.**	**.**	**.**	I	T	K	.	.	I	**.**	.	G	A	**.**	R	**.**	-	-	-	.	.	.	**G**	.	1	1	-	-	2
#8	**.**	**.**	S	S	.	**.**	**.**	**.**	I	T	K	.	.	I	**.**	.	G	.	**.**	R	**.**	-	-	-	K	.	D	**.**	.	1	-	-	-	1
#9	**.**	**.**	S	S	.	**.**	**.**	**.**	I	T	K	.	.	I	**S**	.	G	.	**E**	R	**.**	-	-	-	.	.	D	**.**	.	2	-	-	-	2
#10	**.**	**.**	.	S	.	**.**	**.**	**.**	.	.	K	.	.	I	**.**	.	G	.	**.**	R	**.**	-	-	-	.	.	.	**.**	.	1	1	-	-	2
#11	**.**	**.**	S	S	.	**.**	**.**	**.**	.	.	K	.	.	I	**.**	.	G	.	**.**	R	**.**	-	-	-	.	.	.	**.**	.	1	-	-	-	1
#12	**.**	**.**	S	S	.	**.**	**.**	**.**	I	T	K	.	.	I	**.**	.	G	.	**.**	R	**.**	-	-	-	.	.	.	**.**	.	1	-	-	-	1
#13	**.**	**.**	S	S	E	**.**	**.**	**.**	I	T	K	.	.	I	**.**	K	G	.	**.**	R	**.**	-	-	-	.	.	D	**.**	.	1	4	-	-	5
#14	**.**	**.**	S	S	E	**.**	**.**	**.**	I	T	K	.	.	I	**.**	K	G	.	**.**	R	**.**	-	-	-	K	.	.	**.**	A	2	-	-	-	2
#15	**.**	**.**	S	S	E	**.**	**.**	**.**	I	T	K	.	.	I	**.**	K	G	.	**.**	R	**.**	-	-	-	.	.	.	**.**	.	1	1	-	-	2
#16	**.**	**.**	S	S	.	**.**	**.**	**.**	I	T	K	.	.	I	**.**	.	G	.	**.**	R	**.**	-	-	-	.	.	D	**.**	.	1	1	-	-	2
#17	**.**	**.**	S	S	E	**.**	**.**	**.**	I	T	K	.	.	I	**.**	.	G	.	**.**	R	**.**	-	-	-	K	.	.	**.**	A	2	-	1	-	3
#18	**.**	**.**	S	S	.	**.**	**.**	**.**	I	T	K	.	.	I	**.**	.	G	.	**.**	R	**.**	-	-	-	.	K	D	**.**	.	-	1	-	-	1
#19	**.**	**.**	S	S	.	**D**	**.**	**.**	I	T	.	.	.	I	**R**	.	G	.	**.**	R	**.**	-	-	-	.	.	D	**.**	.	-	1	-	-	1
#20	**.**	**.**	S	S	.	**.**	**.**	**.**	I	T	K	.	.	I	**.**	.	G	.	**.**	R	**.**	-	-	-	K	T	.	**.**	A	-	1	-	-	1
#21	**.**	**.**	S	S	.	**.**	**.**	**.**	I	T	K	.	.	I	**.**	.	G	.	**.**	R	**.**	-	-	-	.	T	D	**.**	.	-	1	-	-	1
#22	**.**	**.**	S	S	.	**.**	**R**	**.**	I	T	.	.	.	I	**.**	K	G	.	**.**	R	**.**	-	-	-	K	.	D	**.**	.	-	1	-	-	1
#23	**.**	**.**	S	S	.	**.**	**.**	**.**	.	.	K	.	.	I	**L**	.	G	.	**.**	R	**.**	-	-	-	.	.	.	**.**	.	-	1	-	-	1
#24	**F**	**.**	S	S	.	**.**	**.**	**.**	.	.	K	.	.	I	**.**	.	G	.	**.**	R	**T**	-	-	-	.	.	.	**.**	.	-	2	-	-	2
#25	**.**	**I**	S	S	.	**.**	**.**	**.**	I	T	K	.	.	I	**.**	K	G	.	**.**	R	**.**	-	-	-	K	.	.	**.**	.	-	1	-	-	1
#26	**.**	**.**	S	S	.	**.**	**R**	**.**	I	T	.	.	.	I	**.**	K	G	.	**.**	R	**.**	-	-	-	.	.	D	**.**	.	-	1	-	-	1
#27	**.**	**.**	S	S	.	**.**	**.**	**.**	I	T	K	K	.	I	**.**	.	G	.	**.**	R	**.**	-	-	-	.	.	D	**.**	A	-	1	-	-	1
#28	**.**	**.**	S	S	.	**.**	**.**	**.**	.	T	K	.	.	I	**.**	.	G	.	**.**	R	**.**	-	-	-	.	.	.	**G**	.	-	-	10	-	10
#29	**.**	**.**	S	S	.	**.**	**.**	**.**	.	T	K	.	.	I	**.**	.	G	.	**.**	R	**.**	-	-	-	.	.	.	**G**	.	-	-	1	-	1
#30	**.**	**.**	.	S	.	**.**	**.**	**.**	.	.	K	.	.	I	**.**	.	G	.	**.**	R	**.**	-	-	-	.	.	.	**G**	.	-	-	8	-	8
#31	**.**	**.**	S	S	E	**.**	**.**	**.**	I	T	K	.	.	I	**.**	.	G	.	**.**	R	**.**	-	-	-	K	.	D	**.**	.	-	-	2	-	2
#32	**.**	**.**	S	S	.	**D**	**.**	**.**	I	T	.	.	.	I	**.**	.	G	.	**.**	R	**.**	-	-	-	K	.	.	**.**	.	-	-	1	-	1
#33	**.**	**.**	.	S	.	**.**	**.**	**.**	.	.	K	.	.	I	**.**	.	G	.	**.**	R	**T**	-	-	-	.	.	.	**G**	.	-	-	1	-	1
#34	**.**	**.**	S	S	.	**.**	**.**	**.**	I	T	.	.	.	I	**L**	.	G	.	**.**	R	**.**	-	-	-	K	.	D	**.**	.	-	-	1	-	1
#35	**.**	**.**	S	S	E	**.**	**.**	**Q**	I	T	K	.	.	I	**.**	K	G	.	**.**	R	**.**	-	-	-	K	.	D	**.**	.	-	-	1	-	1
#36	**.**	**.**	S	S	.	**.**	**.**	**.**	.	.	K	.	T	I	**.**	.	G	.	**.**	R	**.**	-	-	-	.	.	.	**.**	.	-	-	1	-	1
#37	**.**	**.**	S	S	.	**.**	**.**	**.**	I	T	K	.	.	I	**.**	.	G	.	**.**	R	**.**	-	-	-	.	.	.	**G**	.	-	-	1	-	1
#38	**.**	**.**	S	S	.	**.**	**.**	**.**	.	.	K	.	.	I	**.**	.	G	.	**.**	S	**.**	-	-	-	.	.	.	**.**	.	-	-	1	-	1
#39	**.**	**.**	S	S	.	**.**	**.**	**.**	.	.	K	.	.	I	**.**	.	G	.	**.**	R	**T**	-	-	-	.	.	.	**.**	.	-	-	-	15	15
#40	**.**	**.**	S	S	.	**.**	**.**	**.**	.	.	K	.	.	I	**.**	.	G	.	**.**	R	**T**	-	-	-	.	.	.	**.**	.	-	-	-	2	2
#41	**.**	**.**	.	S	.	**.**	**.**	**.**	.	.	K	.	.	I	**.**	.	G	.	**.**	R	**.**	-	-	-	.	.	.	**G**	.	-	-	-	1	1
#42	**.**	**.**	S	S	.	**.**	**.**	**.**	.	.	K	.	.	I	**.**	.	G	.	**.**	R	**.**	-	-	-	K	.	.	**.**	.	-	-	-	10	10
#43	**.**	**.**	S	S	.	**.**	**.**	**.**	.	.	K	.	.	I	**.**	.	G	.	**.**	R	**.**	-	-	-	K	.	.	**.**	.	-	-	-	1	1
#44	**.**	**.**	S	S	.	**.**	**.**	**.**	.	.	K	.	.	I	**.**	.	G	.	**.**	R	**T**	-	-	-	.	.	.	**G**	.	-	-	-	1	1
ProteinStructure*		β1	α3		α4	α4	α4			α5	α5	α5																		31	24	29	30	114

Newly identified amino acid changes are in bold. *Protein secondary structure and segment are after Song, et al. 2012 [51].

The distribution of *PvTRAP* haplotypes in southern Thailand (Yala and Narathiwat provinces) was rather skewed towards few haplotypes as shown by a relatively lower value of haplotype diversity (*h* = 0.653±0.063) whereas parasite populations from Tak, Prachuap Khirikhan and Chanthaburi provinces had significantly higher haplotype diversity values, suggesting a more even distribution of haplotype frequencies in these latter endemic regions (Table 2). It is noteworthy that the predominant haplotypes differed between endemic areas. For examples, haplotypes #39 (n = 15) and #42 (n = 10) were exclusively found in Yala and Narathiwat provinces whereas haplotypes #28 (n = 10) and #30 (n = 8) were unique for Chanthaburi province. In contrast, several haplotypes were shared between isolates from Tak and Prachuap Khirikhan provinces (Table 1). The nucleotide diversity (π) of *PvTRAP* varied from 0.00117±0.00014 (isolates from Yala and Narathiwat provinces) to 0.00272±0.00032 (isolates from Prachuap Khirikhan province). The magnitudes of nucleotide diversity in this locus did not significantly differ among isolates from Tak, Prachuap Khirikhan and Chanthaburi provinces but these were significantly greater than that from Yala and Narathiwat provinces (*p*<10^−4^) (Table 2).

**Table 2:** Variation in the PvTRAP sequences of Thai isolates from diverse endemic areas.

	Tak	Prachuap Khirikhan	Chanthaburi	Yala and Narathiwat	All
N	30	25	29	30	114
M	14	24	18	7	32
S	14	23	18	7	31
H	17	18	12	6	43
*h*±S.D.	0.956±0.018	0.957±0.027	0.818±0.053	0.653±0.063	0.954±0.008
π±S.D.	0.00220±0.00017	0.00272±0.00032	0.00212±0.00032	0.00117±0.00014###	0.00272±0.00009
*d_S_*±S.E. (all)	0.00018±0.00017	0.00074±0.00043	0.00069±0.00052	0.00201±0.00142	0.00118±0.00081
Domain I	0.00000±0.00000	0.00397±0.00467	0.00000±0.00000	0.00000±0.00000	0.00087±0.00097
Domain II	0.00000±0.00000	0.00029±0.00027	0.00048±0.00046	0.00176±0.00130	0.00067±0.00038
Domain III	0.00000±0.00000	0.00000±0.00000	0.00000±0.00000	0.00000±0.00000	0.00000±0.00000
Domain IV	0.00000±0.00000	0.00002±0.00002	0.00000±0.00000	0.00000±0.00000	0.00000±0.00000
Domain V	0.00000±0.00000	0.00000±0.00000	0.00000±0.00000	0.00000±0.00000	0.00000±0.00000
Domain VI	0.00250±0.00281	0.00575±0.00593	0.00721±0.00781	0.01906±0.00000	0.01245±0.01187
*d_N_*±S.E. (all)	0.00278±0.00090**	0.00327±0.00086**	0.00252±0.00077*	0.00091±0.00052	0.00314±0.00090
Domain I	0.00000±0.00000	0.00233±0.00232	0.00000±0.00000	0.00000±0.00000	0.00053±0.00055
Domain II	0.00264±0.00134*	0.00428±0.00161*	0.00406±0.00161*	0.00014±0.00014	0.00396±0.00173
Domain III	0.00423±0.00426	0.00442±0.00439	0.00067±0.00071	0.00000±0.00000	0.00261±0.00266
Domain IV	0.00381±0.00162*	0.00314±0.00132*	0.00248±0.00118*	0.00227±0.00141	0.00373±0.00157*
Domain V	0.00000±0.00000	0.00000±0.00000	0.00000±0.00000	0.00000±0.00000	0.00000±0.00000
Domain VI	0.00000±0.00000	0.00000±0.00000	0.00000±0.00000	0.00000±0.00000	0.00000±0.00000

N, number of sequences; M, number of mutations; S, number of segregating sites; H, number of haplotypes.

Z-tests of the hypothesis that π equals the corresponding value for each population: ###p<0.0001.

Z-tests of the hypothesis that mean *d_S_* equals that of mean *d_N_* : *p<0.05; **p<0.01.

### Population differentiation

**Figure 1:**
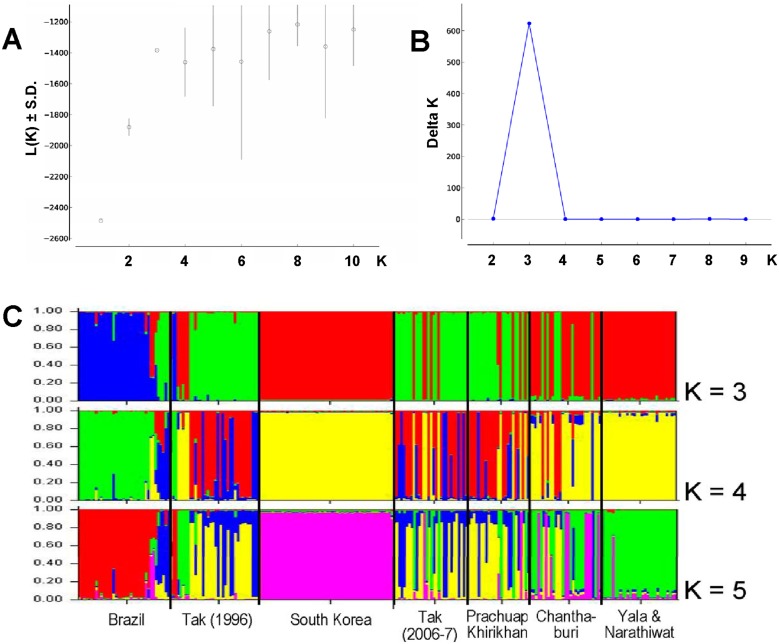
Determination of the most likely number of clusters of *PvTRAP* haplotypes by *ad hoc* methods: (A) relationship between K and mean of estimated mean log likelihood of K and its standard deviation or L(K) ± S.D. [45], (B) relationship between K and delta K [46]. (C) Graphical representation of the genetic structure of populations from Brazil, South Korea and each endemic area of Thailand. Results are given for K3, K4 and K5.

**Figure 2:**
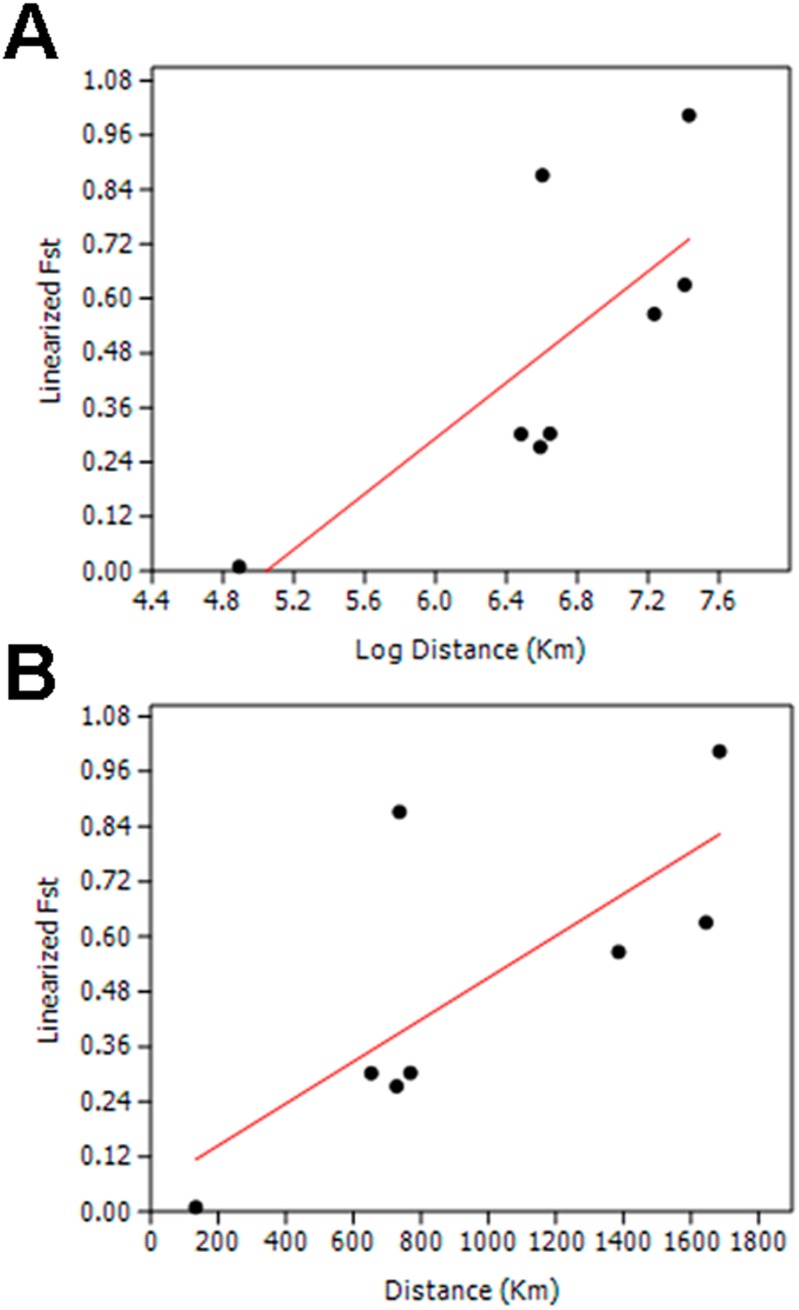
Regression analysis of pairwise linearized *Fst* regressed on natural logarithm geographic distances between *P. vivax* populations in Thailand (A) and pairwise linearized *Fst* regressed on geographic distances between populations (B).

We did not observe population differentiation between isolates from Tak and Prachuap Khirikhan provinces in which the *F_ST_* values did not deviate significantly from zero, implying that they shared a common origin or a high genetic admixture. However, *P. vivax* populations from Tak and Prachuap Khirikhan provinces displayed the *F_ST_* values that were significantly greater than zero when compared with populations from Chanthaburi province (p<10^−5^) as well as that from Yala and Narathiwat provinces (p<10^−5^). A significant departure from neutrality was also noted when isolates from Yala and Narathiwat provinces were compared with those from Chanthaburi province (p<10^−5^), implying genetic differentiation between populations with minimum or absence of gene flow between these areas. Furthermore, the *Fst* values between Tak isolates in the present study (collected in 2006–2007) and those collected in 1996 [19] did not differ significantly (p = 0.23), suggesting genetic stability in *P. vivax* population in this area (Table 3). On the other hand, *P. vivax* populations from Brazil, South Korea and Thailand were significantly different from one another, indicating strong differentiation and reproductive isolation among populations. These results were in line with analysis using STRUCTURE version 2.3.4 software in which the subpopulation likelihood reached a plateau from K = 3 to K = 6. Consistently, a greatest delta K value was also obtained at K = 3 (Figure 1). The genetic structure at K = 3 could differentiate Brazilian isolates from Thai and South Korean parasite populations while a separation of South Korean and southern Thai *P. vivax* populations were not perceived. Similar findings were observed at K = 4. A more concordant results between those obtained by STRUCTURE program and the *Fst* parameters occurred when K = 5 in which isolates from Tak collected during different periods and from Prachuap Khirikhan were indistinguishable whereas South Korean and southern Thai populations were distinct (Figure 1). Regression analysis has shown positive correlation between pairwise linearized *Fst* and the natural logarithm of linear distance between endemic areas (Mantel test, *r* = 0.4619, *p* = 0.024). Likewise, positive correlation was found when geographic linear distance between endemic areas was considered (Mantel test, *r* = 0.4503, *p* = 0.028), implying that the majority of *P. vivax* populations in Thailand were genetically isolated by distance (Figure 2).

**Table 3:** Genetic differentiation (*F_ST_* indices) of *P. vivax* populations based on the PvTRAP locus.

	Tak (2006–7)	Prachuap Khirikhan	Chanthaburi	Yala and Narathiwat	Tak (1996)	Brazil
Prachuap Khirikhan	0.0001					
Chanthaburi	0.2324***	0.2319***				
Yala and Narathiwat	0.5001***	0.4657***	0.3615***			
Tak (1996)	0.0098	0.0003	0.2147***	0.3867***		
Brazil	0.5072***	0.4708***	0.4380***	0.4603***	0.4325***	
Korea	0.7057***	0.6907***	0.5514***	0.7305***	0.5742***	0.5630***

***p<0.00001.

### Tests for departure from neutral evolution

**Figure 3:**
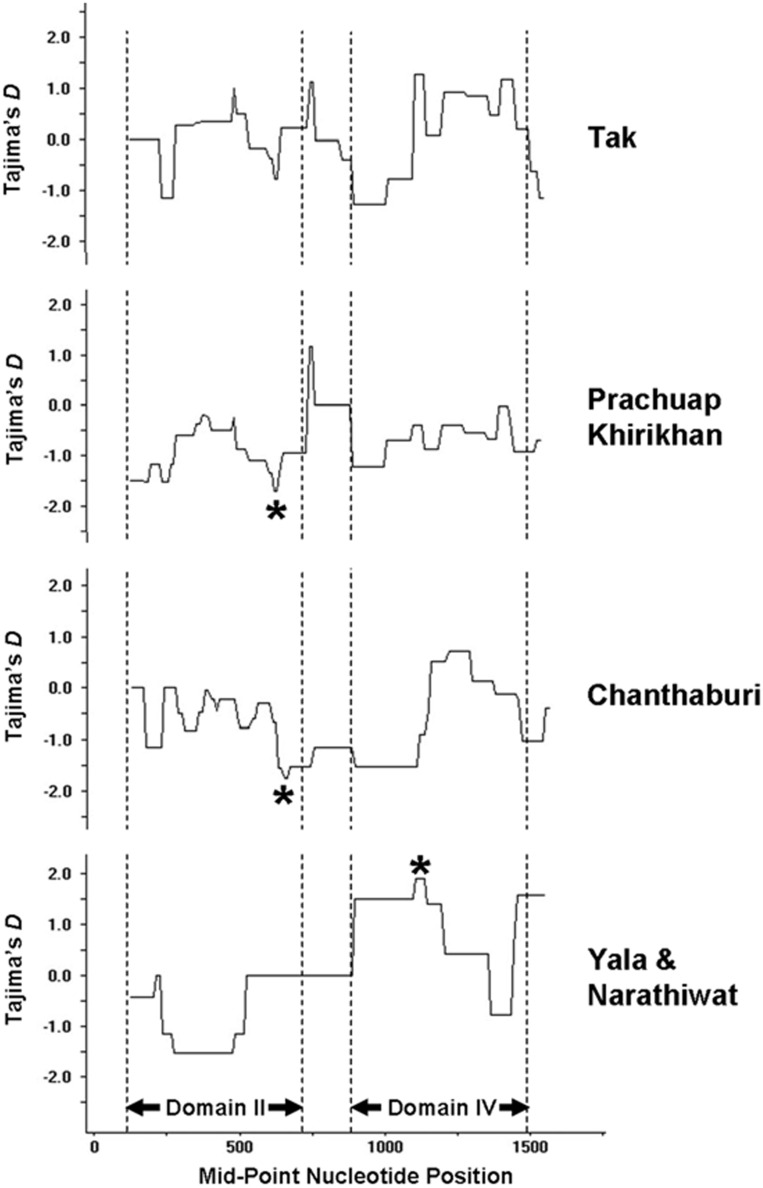
Sliding window plots of Tajima’s *D* across *PvTRAP* sequences from each population in Thailand. Asterisks denote values with *p*<0.05. Plots are based on a window size of 250 bp and a step size of 10 bp.

Analysis of patterns of nucleotide substitutions in the *PvTRAP* sequences of isolates in this study reveals that the rate of nonsynonymous substitutions per nonsynonymous sites or *d_N_* significantly exceeds that of synonymous substitutions per synonymous sites or *d_S_* in all parasite populations (*p*<0.05 or *p*<0.01) except *P. vivax* isolates from Yala and Narathiwat provinces (Table 2). In contrast, *d_S_* was greater than *d_N_* for isolates from Yala and Narathiwat provinces although the difference was not statistically meaningful (*p* = 0.467). Closer looks into the rate of nucleotide substitutions in each domain of *PvTRAP* has revealed that *d_N_* significantly outnumbered *d_S_* in domains II and IV of isolates from Tak, Prachuap Khirikhan and Chanthaburi provinces. Although no significant difference in *d_S_* and *d_N_* for the entire coding region of the *PvTRAP* gene was observed when all parasite populations were considered, *d_N_* in domain IV was obviously greater than *d_S_* (Table 2). Meanwhile, statistics based on Tajima’s *D* and Fu & Li’s D* and F* did not show evidences of departure from neutral expectation (Table 4). However, sliding window analysis reveals significant negative Tajima’s *D* in domain II of *P. vivax* populations from Prachuap Khirikhan and Chanthaburi provinces, suggesting purifying selection at certain residues in this gene. In contrast, evidence of balancing selection was detected in domain IV of Yala and Narathiwat isolates as shown by significant positive *D* values. Meanwhile, no departure from neutrality was detected in malaria population from Tak province (Figure 3).

**Table 4:** Intraspecific and interspecific neutrality tests for the PvTRAP locus from each endemic area of Thailand.

Province/Endemic area	Tajima’s *D*	Fu & Li’s D*	Fu & Li’s F*	Out group: *P. cynomolgi*		Out group: *P. knowlesi*	
				Fu & Li’s *D*	Fu & Li’s *F*	Fu & Li’s *D*	Fu & Li’s *F*
Tak (n = 30)	0.097	−0.143	−0.080	0.431	0.350	0.987	1.067
Prachuap Khirikhan (n = 25)	−0.967	−0.686	−0.905	−0.086	−0.401	0.284	0.025
Chanthaburi (n = 29)	−0.818	−1.429	−1.451	−0.490	−0.798	−0.080	−0.383
Yala and Narathiwat (n = 30)	0.278	−0.120	0.000	0.010	0.227	0.427	0.591
All (n = 114)	−0.698	−1.714	−1.572	−1.055	−1.120	−0.534	−0.553

All values did not have significance departure from zero.

Results from interspecific comparison between *PvTRAP* and *PcyTRAP* using the MacDonald-Kreitman test has shown that each parasite population except that from Yala and Narathiwat provinces had significant deviation in positive direction from neutral expectation (*p*<0.01), reflecting a signature of positive or balancing selection. Consistent results were obtained when the TRAP gene of *P. knowlesi* was used as the outgroup sequence (Table 5). In contrast, application of the Fu & Li’s *D* and *F* tests using either *PcyTRAP* or *PkTRAP* as outgroup sequences did not yield significant departure from neutrality. Meanwhile, positively selected sites were identified at 4 codons in domain II and 3 codons in domain IV by one or more of the methods implemented in the HyPhy package (Table 6). Likewise, negatively selected sites were detected in residue 21 in domain I, residues 30, 100, 133 in domain II and residues 519 and 539 in domain VI, suggesting functional constraint occurring at certain residues in this protein.

**Table 5:** McDonald–Kreitman tests on TRAP of *Plasmodium vivax* from diverse geographic origins with *P. cynomolgi* orthologue as outgroup species.

Population	Polymorphic changes within *P. vivax*		Fixed differences between *species*		Neutrality index	*p* value
	Synonymous	Nonsynonymous	Synonymous	Nonsynonymous		
Tak	1	13	116	140	10.77	**0.0045**
Prachuap Khirikhan	3	21	117	138	5.93	**0.0011**
Chanthaburi	2	16	117	140	6.69	**0.0053**
Yala and Narathiwat	3	4	115	145	1.06	1.0000
All	6	23	117	136	3.298	**0.0096**

Note: Repeats are excluded from all analyses.

**Table 6:** Codon-based analysis of departure from neutrality in the PvTRAP locus of Thai isolates.

Domain	Codon	SLAC		FEL		REL		MEME		Consensus*			
		*d_N_ - d_S_*	*p* value	*d_N_ - d_S_*	*p* value	*d_N_ - d_S_*	Bayes Factor	ω+	*p* value	SLAC	FEL	REL	MEM
II	120	93.598	0.213	2821.14	0.091	73.567	9897.75	>100	0.115		+	+	
II	134	74.642	0.496	2675.58	0.208	73.381	195736	>100	0.230			+	
II	206	84.790	0.280	3760.95	0.105	73.323	702283	>100	0.051			+	+
II	255	149.777	0.112	3884.15	0.063	73.501	2.27475×10^10^	>100	0.084		+	+	+
IV	410	95.051	0.401	3087.63	0.178	73.377	8780900	>100	0.203			+	
IV	421	55.136	0.594	1821.01	0.252	73.389	2484.53	>100	0.267			+	
IV	439	83.134	0.304	2728.63	0.104	73.541	267258	>100	0.129			+	
I	21	−102.062	0.151	−2637.21	0.063	−38.418	35812.5	<100	>0.100		-	-	
II	30	−204.123	0.023	−5228.32	0.011	−38.410	29828.7	<100	>0.100	-	-	-	
II	100	−46.299	0.333	−1399.13	0.131	−38.407	29051.4	<100	>0.100			-	
II	133	−31.970	0.483	−1019.35	0.210	−38.100	8558.41	<100	>0.100			-	
VI	519	−92.599	0.111	−2873.15	0.032	−38.407	29054.6	<100	>0.100		-	-	
VI	539	−83.719	0.199	−3571.07	0.051	−38.391	27525.8	<100	>0.100		-	-	

*positively selected site, +; negatively selected site, -.

Default significance levels in Datamonkey program: SLAC, *p*<0.1; FEL, *p*<0.1; REL, Bayes Factor>1000; MEME, *p*<0.1.

### Amino acid substitutions and predicted HLA binding

The amino acid substitutions in PvTRAP of Thai isolates had slightly higher percentage of conservative changes with respect to polarity and charge property as 67.7% and 58.1% of these substitutions, respectively, were unchanged. Similar percentages of these changes were observed among worldwide isolates, being 64.6% and 56.9%, respectively (Table S2). Closer look into amino acid substitutions and potential HLA-binding peptides as predicted by the high scores for the C-terminal cleavage and the transporter associated with antigen processing efficiency [48] have shown that a number of substituted residues have remarkably reduced predicted scores; thereby, the property of these epitopes could be altered. It is noteworthy that substituted residues in several of these potential epitopes occurred in domains II and IV of PvTRAP (Table 6).

### Intragenic recombination

Evidence of recombination in the *PvTRAP* locus analyzed by using the HyPhy package has identified evidence of 1 recombination breakpoint with significant topological incongruence between AICc score of the best fitting GARD model (*p*<0.05) for *P. vivax* populations in Thailand except those from Yala and Narathiwat provinces (0.05<*p*<0.1). Although these recombination breakpoints occur at different positions, i.e. between nucleotides 1008 and 1009, 1170 and 1171, and 1173 and 1174 for Chanthaburi, Prachuap Khirikhan and Tak provinces, respectively, it is noteworthy that all were located in domain IV of the *PvTRAP* gene. Meanwhile, estimation of the parameter Rm reveals 3 and 5 recombination sites in parasite populations from Tak and Prachuap Khirikhan provinces whereas Chanthaburi isolates and southern Thai *P. vivax* populations had equal number of potential recombination sites (Rm = 2). Locations of potential recombination sites for each population are listed in Table S3, most of which span domain IV. These results suggest that intragenic recombination could shape diversity at the *PvTRAP* locus.

### Phylogenetic relationship

**Figure 4:**
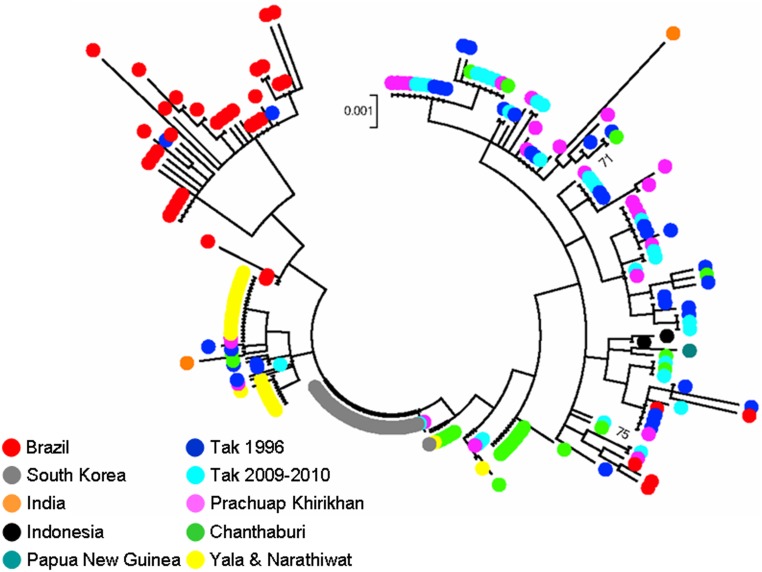
Phylogenetic analysis inferred from a total of 246 PvTRAP sequences from this study and those previously reported [19], [28] using the Maximum Likelihood method based on the Tamura 3-parameter model. Gaps and missing data are excluded from analysis. Bootstrap values more than 70% are shown. Dots with different colors represent isolates and their geographic origins as listed on the lower left.

Model test reveals that the Tamura 3-parameter with the rate variation model that allowed for some sites to be evolutionarily invariable gave the lowest BIC value. The tree with the highest log likelihood (−3285.15) reveals that the majority of Brazilian isolates were placed in a separate lineage while the majority of Tak isolates collected in 1996, in 2006–2007 and most Prachuap Khirikhan isolates were clustered together. Several isolates from Chanthaburi, Yala and Narathiwat provinces seem to be located in separate branches from the remaining Thai isolates. However, there was no clear lineage for each parasite population because several isolates from different endemic areas shared or was placed in closely related branches. More importantly, all branches in this phylogenetic inference received bootstrap values less than 80%, suggesting no distinct lineage of the *PvTRAP* haplotypes from worldwide origins (Figure 4).

## Discussion

Host adhesion and motility are fundamental properties of malarial sporozoites to establish infection that involves sporozoite-specific proteins, one of which is TRAP. It has been shown that TRAP-deficient sporozoites were severely impaired in their host cell adherence property and gliding motility; thereby, invasion of mosquito’s salivary glands and hepatocytes was interrupted [14], [49]. Two extracellular portions of malarial TRAP, the von Willebrand A-domain and the thrombospondin repeats located in domains II and IV, respectively, are crucial for initial host cell adherence and stabilization of adhesion/deadhesion during gliding mobility of sporozoites [49], [50]. Crystal structure analyses spanning the von Willebrand A-domain and the thrombospondin repeats of PvTRAP and PfTRAP reveal two conformational states, open and closed structures. Such structural phase transition is possibly responsible for ‘stick-and-slip’ or gliding motility of sporozoites through the actomyosin motility apparatus [51], [52]. The sequence motif of metal-ion-dependent adhesion sites (MIDAS) in von Willebrand A-domain and the extensible β ribbon that implicates in ligand binding during the open high-affinity state [51] were perfectly conserved in all PvTRAP haplotypes as shown in this study. Although amino acid substitutions were observed in the β1 and α3 domains, charge and polarity of these substituted residues were retained, suggesting structural constraint in these portions of the protein (Table 1). Experimental studies have shown that artificially engineered mutations of the MIDAS-coordinating amino acid residues that were conserved across malaria species could impair gliding motility and sporozoite infectivity to both mosquito and mammalian hosts [49], [50]. On the other hand, alteration in both charge and polarity profiles of some substituted amino acids (residues 143, 166, 172 and 176, Tables 1 and S2) were identified in α4 and α5 domains, implying that changes in these residues may not drastically affect functional conformation of PvTRAP. Nevertheless, further experimental studies are required to address this issue.

The extent of sequence diversity of the *PvTRAP* locus among parasite populations in Thailand seems to be comparable among endemic areas except that from southern region (Table 2). The results from this study are in line with our recent analyses of other major vaccine antigens, i.e. apical membrane antigen-1 (Pvama-1), merozoite surface protein-1 (Pvmsp-1), merozoite surface protein-4 (Pvmsp-4) and merozoite surface protein-5 (Pvmsp-5) of *P. vivax* showing that southern parasite population possesses significantly lower levels of nucleotide diversity, number of haplotypes and haplotype diversity than those of northwestern parasite population [24], [25], [53]. Heterogeneity in the *PvTRAP* sequences could have been arisen from selective pressures and intragenic recombination. Recombination confers evolutionary advantages because creation of adaptive traits or removal of deleterious mutants is enabled. Importantly, most recombination sites were identified within or spanning domain IV of the *PvTRAP* locus where signature of positive selection was identified. The relatively higher levels of nucleotide diversity of *PvTRAP* from Thai-Mynmar border (Tak and Prachuap Khirikhan isolates) than that from Thai-Cambodia border (Chanthaburi isolates) seems to be in line with Rm in each population. It is likely that transmigration of people could maintain and spread of *P. vivax* harboring variant *PvTRAP* haplotypes while recombination could further generate novel alleles. Intriguingly, it seems that reduction in genetic diversity of southern parasite population may not be simply explained by clonal population structure of *P. vivax* in this region because recombination event could be traced despite the paucity of potential recombination sites observed (Rm = 2). In contrast, several decades of extensive malaria control in this region and a lack of remarkable re-introduction of malaria cases from Malaysia could have resulted in population bottleneck as previously suggested [24]. Therefore, after bottleneck effect and relaxation of malaria control in southern Thailand because of difficulty in implementation due to local political unrest, it could be possible that recombination between different *PvTRAP* haplotypes may gradually increase the level of genetic diversity in this population.

Our analyses have shown that domains II and IV of the *PvTRAP* locus had significant difference in the rate of nonsynonymous substitutions than that of synonymous substitutions. Evidence of positive or balancing selection was also reaffirmed by the McDonald-Kreitman test. Although departure from neutrality in the entire coding PvTRAP sequence was not apparent by using Tajima’s *D* and related statistics, sliding window analysis has identified significant negative Tajima’s *D* values in domains II of *P. vivax* population from Prachuap Khirikhan and Chanthaburi provinces while significant positive values occurred in domain IV among isolates from Yala and Narathiwat provinces. Differential patterns of departure from neutrality among populations could reflect different evolutionary forces exerted on PvTRAP of each population. Alternatively, intrinsic statistical property of Tajima’s *D* statistics can be confounded by demographic processes [54]. Therefore, significant positive Tajima’s *D* observed in southern Thai isolates could imply balancing selection or a recent bottleneck because an excess of intermediate frequency alleles as rare alleles are lost from the population due to genetic drift can be found immediately after the bottleneck. Meanwhile, significant negative Tajima’s *D* value in domains II could imply purifying selection and selective sweep while migration and population growth may be the confounders [55]. Nevertheless, positively selected codons were found based on analyses using SLAC, FEL, REL and MEME methods while negatively selected residues were detected in domains I, II and VI (Table 6). Taken together, it seems that co-existence of both negatively selected amino acids and residues under positive selection could stem from functional constraint of this molecule and probably driven by host immune pressure, respectively. Meanwhile, it seems that intragenic recombinantion was pronounced in populations from Tak and Prachuap Khirikhan provinces while relatively few recombinations occurred in parasites from Chanthaburi province and southern isolates. These findings are in accord with our previous analysis on *PvMSP4* and *PvMSP5* in which frequency of recombination seems to correlate with endemicity of each area [25], [53].

Despite low level of sequence diversity, genetic distances inferred from the *PvTRAP* locus among *P. vivax* populations could be deployed for analysis of population structure. Pairwise comparison of populations revealed that South Korean isolates displayed high and significant *Fst* indices when compared with malaria populations in other endemic areas. Likewise, slightly lower but significant *Fst* indices were observed when isolates from Brazil were compared with populations elsewhere (Table 3). Therefore, gene flow between *P. vivax* populations in Thailand, Brazil and Korea may not occur because geographic distance has precluded intra- and intercontinental spread of parasites. Although malaria can be transferred between remote endemic areas through infected travelers, this situation could be inefficient due to rarity in numbers of such cases to contribute to genetic admixture of parasites in these three countries. However, closer look into genetic differentiation between 4 major malaria endemic areas of Thailand, significant genetic differentiation of *P. vivax* population between endemic areas was observed except parasites from Tak province collected over a decade apart (1996 and 2006–2007) having low and non-significant *Fst* indices. Therefore, diversity in the *PvTRAP* locus of parasites in Thailand seems to exhibit spatial but not temporal variation. It is noteworthy that regression analysis of linear geographic as well as normal logarithm of geographic distances and pairwise linearized *Fst* values yielded significant correlation (Mantel test, *p* = 0.024 and 0.028, respectively), indicating that *P. vivax* populations in these 4 endemic areas have been isolated by distance (Figure 2). Therefore, it seems likely that the flight range of mosquito vectors was the major determinant for magnitude of genetic differentiation of *P. vivax* populations between endemic areas. However, there remain some other factors that could influence and shape genetic structure of malaria in Thailand. Although geographic distance between Tak and Prachuap Khirikhan provinces is about 160 kilomerters longer than that between Tak and Chanthaburi provinces, significant genetic differentiation occurred in the latter but not the former. It should be noted that transmigration of people along Thai-Myanmar border where both Tak and Prachuap Khirikhan provinces are located is intense during the past decades whereas transmigration of gem miners who, not uncommonly, carried malaria parasites in their circulation between Tak and Chanthaburi provinces had ceased for over 3 decades ago [56]. Furthermore, the abundance of malaria vectors along Thai-Myanmar border could facilitate gene flow of *P. vivax* in these areas. In contrast, ecological niches of *Anopheles* vectors between Tak and Chanthaburi provinces are largely interrupted by focal deforestation and urbanization, precluding natural spread of malaria between these areas.

Analysis of hierarchical splitting of gene pools by STRUCTURE has shown that K = 3 is an optimum value. Intriguingly, K = 3 could not subdivide South Korean and southern Thai isolates that in fact differed biologically and genetically. A very long incubation period of vivax malaria from 230 to 300 days has been recognized among South Korean isolates [28] but, to our knowledge, has never been recorded in Thai strains. Furthermore, phylogenetic analysis has placed all South Korean isolates to a separate cluster from southern Thai isolates, *albeit* without significant bootstrap support (Figure 3). In contrast, the partition at K = 5 is more consistent with results from analysis using *Fst* indices in which distinct subpopulation structure was perceived between isolates from southern Thailand and those from South Korea whilst at K<5, no discernible difference was observed between these populations. Meanwhile, phylogenetic inference does not support distinct clusters of isolates relating to their geographic origins because no branch received remarkably high bootstrap values, consistent with our previous study [19]. Therefore, genetic diversity of the *PvTRAP* locus displays tendency towards geographic variation (Figure 4).

Protective immunity against malarial TRAP involves both humoral and cell mediated immune responses. Antibodies against TRAP targeting domains involved in gliding motility and hepatocyte binding ligands could prevent sporozoite invasion into hepatocytes [13] while TRAP-specific cytotoxic T cells could destroy sporozoite-infected hepatocytes [57]; thereby, exoerythrocytic development does not ensue. The roles of anti-TRAP antibodies in partial protection from or significantly reduced risk of falciparum malaria have been demonstrated in African endemic areas [12], [58], [59]. With analogy to the thrombospodin-related motif (TRM) of PfTRAP that binds heparin sulfate proteoglycan of hepatocyte, the TRM in domain III of PvTRAP is relatively conserved with two amino acid substitutions, i.e. WTACSVTCGR(or K)GTH (or Q) SRSR, while the MIDAS motif is perfectly conserved among clinical isolates. Therefore, limited diversity at the functional domains in PvTRAP has encouraged vaccine incorporation. Meanwhile, several CD4+ and CD8+ T cell epitopes have been mapped across PfTRAP, some of which have been associated with protection [57], [60], [61]. Importantly, T cell responses to PfTRAP were commonly allele-specific although certain epitopes could induce or serve as targets of cross-reactive immunity [60]. Recent studies have highlighted the importance of protective roles of CD8+ T cell and memory T cell responses to PfTRAP from clinical malaria [61], [62]. The significance of diversity in these T cell epitopes among natural malaria population remains to be elucidated in term of vaccine efficacy. To date, little is known about immunological responses to PvTRAP although vaccination study using synthetic peptide encompassing hepatocyte-binding ligand of the protein has conferred protection against parasite challenge in *Aotus* monkeys [63]. Our analysis suggests that polymorphisms in PvTRAP could affect T cell recognition because amino acid substitutions in several predicted HLA-binding peptides have remarkably altered the predicted epitope scores (Table 7).

**Table 7:** Amino acid substitutions in potential T cell epitopes of PvTRAP and predicted scores*.

HLA	Domain	Predicted epitopes	Predicted score	%Reduction	No. isolates
A1	I	YLLVVF**L**LY	2.1645		112
		YLLVVF**F**LY	1.6240	24.97	2
A1	II	VCNESVDL**Y**	0.9376		113
		VCNESVDL**I**	0.7571	19.25	1
A2	I	F**L**LYVSIFA	1.1395		112
		F**F**LYVSIFA	0.5416	52.47	2
A2	II	NMTAAL**EE**V	1.1851		47
		NMTAAL**DE**V	1.1104	6.30	65
		NMTAAL**DD**V	0.7819	34.02	2
A3	II	Y**T**ALEVAKK	0.8093		63
		Y**R**ALEVAKK	0.2512	68.96	51
A3	II	KVTELRK**S**Y	0.7907		102
		KVTELRK**T**Y	0.5974	24.45	12
A24	I	VF**L**LYVSIF	1.7214		112
		VF**F**LYVSIF	1.4858	13.69	2
A26	II	ELRK**T**YSPY	2.0070		12
		ELRK**S**YSPY	1.9132	4.67	102
		ESVD**L**YLLV	1.3830	31.09	1
		ESVD**I**YLLV	1.3656	31.96	113
B7	II	KLK**Q**RNV**S**L	0.8786		112
		KLK**K**RNV**S**L	0.8400	4.39	1
		KLK**Q**RNV**T**L	0.7263	17.33	1
B7	II	K**Q**RNV**T**LAV	0.9688		1
		K**K**RNV**S**LAV	0.8745	9.73	1
		K**Q**RNV**S**LAV	0.7908	18.37	112
B7	IV	LPVPAPLP**A**	1.3094		2
		LPVPAPLP**T**	1.1464	12.45	112
B8	II	RPRE**R**NCKF	1.9566		1
		RPRE**L**NCKF	1.3892	29.00	2
		RPRE**S**NCKF	1.0950	44.04	2
		RPRE**P**NCKF	1.0440	46.64	109
B8	II	ELRK**T**YSPY	0.9655		12
		ELRK**S**YSPY	0.8494	12.02	102
B27	II	**K**RNV**S**LAVI	1.2095		1
		**Q**RNV**S**LAVI	0.9591	20.70	112
		**Q**RNV**T**LAVI	0.9591	20.70	1
B27	II	K**Q**RNV**S**LAV	0.7781		112
		K**Q**RNV**T**LAV	0.7781	0	1
		K**K**RNV**S**LAV	0.5384	30.81	1
B27	III	G**R**GTHSRSR	1.2775		96
		G**K**GTHSRSR	0.4904	61.61	18
B39	I	SYLLVVF**L**L	0.7915		112
		SYLLVVF**F**L	0.6755	14.66	2
B44	IV	NEKVIP**N**PL	1.4650		110
		NEKVIP**T**PL	1.4469	1.24	3
		NEKVIP**K**PL	1.2157	17.02	1
B58	I	KSYLLVVF**F**	1.4989		2
		KSYLLVVF**L**	0.8347	44.31	112
B62	II	K**Q**RNV**T**LAV	1.1504		1
		K**Q**RNV**S**LAV	1.1443	0.53	112
		K**K**RNV**S**LAV	0.2828	75.42	1

*Based on the C-terminal cleavage and the transporter associated with antigen processing efficiency [48].

In conclusion, our analysis has shown that genetic diversity in the *PvTRAP* locus of Thai *P. vivax* isolates exhibits geographic variation and population structure. Positive selection and intragenic recombination have shaped diversity at the PvTRAP locus. The low level of sequence diversity in PvTRAP among clinical isolates as shown in this study may not drastically compromise vaccine design.

## Supporting Information

Table S1 **Haplotypes of PvTRAP from Thai isolates.**
(DOC)

Table S2 **Characteristics of amino acid substitutions in PvTRAP of worldwide and Thai isolates.**
(DOC)

Table S3 **Minimum number of recombination events (Rm) and recombination sites in the PvTRAP gene of each parasite population in Thailand.**
(DOC)
