# Correction: Spatial variation in genetic diversity and natural selection on the thrombospondin-related adhesive protein locus of *Plasmodium vivax (PvTRAP)*

**DOI:** 10.1371/journal.pone.0203163

**Published:** 2018-08-23

**Authors:** Rattiporn Kosuwin, Chaturong Putaporntip, Hiroshi Tachibana, Somchai Jongwutiwes

One of the affiliations for the first author is missing. Rattiporn Kosuwin is also affiliated with: Inter-Department Program of Biomedical Sciences, Faculty of Graduate School, Chulalongkorn University, Bangkok, Thailand.
