# Supplementary material for: Spatial Variation in Genetic Diversity and Natural Selection on the Thrombospondin-Related Adhesive Protein Locus of Plasmodium vivax (PvTRAP)
Source: PLoS One. 2014 Oct 21;9(10):e110463. doi: 10.1371/journal.pone.0110463 (PMC4204863; doi:10.1371/journal.pone.0110463)
Supplement: Table S1 — Haplotypes of PvTRAP from Thai isolates. (DOC) [file pone.0110463.s001.doc]

**Table S1** Haplotypes of *PvTRAP* from Thai isolates

| **Haplotype** | **n** |  |  |  |  |  |  |  |  |  |  |  |  |  |  |  |  |  |  |  |  |  | **1** | **1** | **1** | **1** | **1** | **1** | **1** | **1** | **1** | **1** | **1** | **1** | **1** | **1** | **1** | **1** | **1** | **1** | **1** |
| --- | --- | --- | --- | --- | --- | --- | --- | --- | --- | --- | --- | --- | --- | --- | --- | --- | --- | --- | --- | --- | --- | --- | --- | --- | --- | --- | --- | --- | --- | --- | --- | --- | --- | --- | --- | --- | --- | --- | --- | --- | --- |
|  |  |  |  |  | **1** | **3** | **3** | **3** | **4** | **4** | **4** | **4** | **4** | **4** | **5** | **5** | **5** | **6** | **6** | **7** | **8** | **9** | **0** | **0** | **1** | **1** | **1** | **1** | **1** | **1** | **1** | **1** | **1** | **2** | **2** | **2** | **3** | **3** | **4** | **5** | **6** |
|  |  | **4** | **6** | **9** | **3** | **0** | **5** | **9** | **0** | **0** | **1** | **2** | **7** | **9** | **1** | **2** | **3** | **1** | **1** | **6** | **8** | **7** | **0** | **1** | **5** | **5** | **5** | **5** | **6** | **6** | **6** | **6** | **6** | **3** | **7** | **7** | **2** | **2** | **0** | **6** | **2** |
|  |  | **3** | **3** | **0** | **3** | **0** | **8** | **7** | **2** | **5** | **0** | **8** | **8** | **7** | **6** | **6** | **9** | **6** | **7** | **4** | **9** | **2** | **8** | **9** | **6** | **7** | **8** | **9** | **0** | **1** | **2** | **3** | **4** | **9** | **1** | **2** | **5** | **7** | **0** | **6** | **6** |
| #1 | 7 | C | C | C | T | C | T | C | A | A | A | G | A | C | A | C | G | C | C | G | A | T | G | A |  |  |  |  |  |  |  |  |  | C | A | C | A | A | A | T | A |
| #2 | 3 | C | C | C | T | C | T | C | T | A | A | G | A | C | A | C | G | C | C | A | A | T | G | A |  |  |  |  |  |  |  |  |  | C | A | C | A | A | A | T | A |
| #3 | 2 | C | C | C | T | C | T | C | A | A | A | G | A | C | A | C | G | C | C | A | A | T | G | A |  |  |  |  |  |  |  |  |  | C | A | C | G | G | A | T | A |
| #4 | 1 | C | C | C | T | C | T | C | A | A | A | G | A | C | A | C | G | C | C | G | A | T | G | A |  |  |  |  |  |  |  |  |  | A | C | C | G | A | C | T | A |
| #5 | 5 | C | C | C | T | C | T | C | A | A | A | G | A | C | A | C | G | C | C | G | A | T | G | A | C | C | A | G | A | T | A | G | C | A | A | C | A | A | A | T | A |
| #6 | 4 | C | C | C | T | C | T | C | T | A | A | G | G | G | A | C | G | C | C | G | A | T | G | A |  |  |  |  |  |  |  |  |  | C | A | C | G | G | A | T | A |
| #7 | 2 | C | C | C | T | C | T | C | A | A | A | G | A | C | A | C | G | C | C | G | G | T | G | A |  |  |  |  |  |  |  |  |  | C | A | C | G | G | A | T | A |
| #8 | 1 | C | C | C | T | C | T | C | T | A | A | G | A | C | A | C | G | C | C | G | A | T | G | A |  |  |  |  |  |  |  |  |  | A | A | C | A | A | A | T | A |
| #9 | 2 | C | C | C | T | C | T | C | T | A | A | G | A | C | A | C | G | T | C | G | A | A | G | A |  |  |  |  |  |  |  |  |  | C | A | C | A | A | A | T | A |
| #10 | 2 | C | C | C | T | C | A | C | T | A | A | G | G | G | A | C | G | C | C | G | A | T | G | A |  |  |  |  |  |  |  |  |  | C | A | C | G | A | A | T | A |
| #11 | 1 | C | C | C | T | C | T | C | T | A | A | G | G | G | A | C | G | C | C | G | A | T | G | A |  |  |  |  |  |  |  |  |  | C | A | C | G | A | A | T | A |
| #12 | 1 | C | C | C | T | C | T | C | T | A | A | G | A | C | A | C | G | C | C | G | A | T | G | A |  |  |  |  |  |  |  |  |  | C | A | C | G | A | A | T | G |
| #13 | 5 | C | C | C | T | C | T | C | A | A | A | G | A | C | A | C | G | C | C | A | A | T | G | A |  |  |  |  |  |  |  |  |  | C | A | C | A | A | A | T | A |
| #14 | 2 | C | C | C | T | C | T | C | A | A | A | G | A | C | A | C | G | C | C | A | A | T | G | A |  |  |  |  |  |  |  |  |  | A | A | C | G | A | C | T | A |
| #15 | 2 | C | C | C | T | C | T | C | A | A | A | G | A | C | A | C | G | C | C | A | A | T | G | A |  |  |  |  |  |  |  |  |  | C | A | C | G | A | A | T | A |
| #16 | 2 | C | C | C | T | C | T | C | T | A | A | G | A | C | A | C | G | C | C | G | A | T | G | A |  |  |  |  |  |  |  |  |  | C | A | C | A | A | A | T | A |
| #17 | 3 | C | C | C | T | C | T | C | A | A | A | G | A | C | A | C | G | C | C | G | A | T | G | A |  |  |  |  |  |  |  |  |  | A | A | C | G | A | C | T | A |
| #18 | 1 | C | C | C | T | C | T | C | T | A | A | G | A | C | A | C | G | C | C | G | A | T | G | A |  |  |  |  |  |  |  |  |  | C | A | A | A | A | A | T | A |
| #19 | 1 | C | C | C | T | C | T | C | T | T | A | G | A | C | T | C | G | C | G | G | A | T | G | A |  |  |  |  |  |  |  |  |  | C | A | C | A | A | A | T | A |
| #20 | 1 | C | C | C | T | C | T | C | T | A | A | G | A | C | A | C | G | C | C | G | A | T | G | A |  |  |  |  |  |  |  |  |  | A | C | C | G | A | C | T | A |
| #21 | 1 | C | C | C | T | C | T | C | T | A | A | G | A | C | A | C | G | C | C | G | A | T | G | A |  |  |  |  |  |  |  |  |  | C | C | C | A | A | A | T | A |
| #22 | 1 | C | T | C | T | C | T | C | T | A | G | G | A | C | T | C | G | C | C | A | A | T | G | A |  |  |  |  |  |  |  |  |  | A | A | C | A | A | A | T | A |
| #23 | 1 | C | C | C | T | C | T | C | T | A | A | G | G | G | A | C | G | T | T | G | A | T | G | A |  |  |  |  |  |  |  |  |  | C | A | C | G | A | A | T | A |
| #24 | 2 | T | C | C | T | C | T | C | T | A | A | G | G | G | A | C | G | C | C | G | A | T | G | C |  |  |  |  |  |  |  |  |  | C | A | C | G | A | A | C | A |
| #25 | 1 | C | C | C | A | C | T | C | T | A | A | G | A | C | A | C | G | C | C | A | A | T | G | A |  |  |  |  |  |  |  |  |  | A | A | C | G | A | A | T | A |
| #26 | 1 | C | C | C | T | C | T | C | T | A | G | G | A | C | T | C | G | C | C | A | A | T | G | A |  |  |  |  |  |  |  |  |  | C | A | C | A | A | A | T | A |
| #27 | 1 | C | C | C | T | C | T | C | T | A | A | G | A | C | A | A | G | C | C | G | A | T | G | A |  |  |  |  |  |  |  |  |  | C | A | C | A | A | C | T | A |
| #28 | 10 | C | C | C | T | C | T | C | T | A | A | G | G | C | A | C | G | C | C | G | A | T | G | A |  |  |  |  |  |  |  |  |  | C | A | C | G | G | A | T | A |
| #29 | 1 | C | C | C | T | T | T | C | T | A | A | G | G | C | A | C | G | C | C | G | A | T | G | A |  |  |  |  |  |  |  |  |  | C | A | C | G | G | A | C | A |
| #30 | 8 | C | C | C | T | C | A | C | T | A | A | G | G | G | A | C | G | C | C | G | A | T | G | A |  |  |  |  |  |  |  |  |  | C | A | C | G | G | A | T | A |
| #31 | 2 | C | C | C | T | C | T | C | A | A | A | G | A | C | A | C | G | C | C | G | A | T | G | A |  |  |  |  |  |  |  |  |  | A | A | C | A | A | A | T | A |
| #32 | 1 | C | C | C | T | C | T | C | T | T | A | G | A | C | T | C | G | C | C | G | A | T | G | A |  |  |  |  |  |  |  |  |  | A | A | C | G | A | A | T | A |
| #33 | 1 | C | C | C | T | C | A | C | T | A | A | G | G | G | A | C | G | C | C | G | A | T | G | C |  |  |  |  |  |  |  |  |  | C | A | C | G | G | A | T | A |
| #34 | 1 | C | C | C | T | C | T | C | T | A | A | G | A | C | T | C | G | C | T | G | A | T | G | A |  |  |  |  |  |  |  |  |  | A | A | C | A | A | A | T | A |
| #35 | 1 | C | C | C | T | C | T | C | A | A | A | A | A | C | A | C | G | C | C | A | A | T | G | A |  |  |  |  |  |  |  |  |  | A | A | C | A | A | A | T | A |
| #36 | 1 | C | C | C | T | C | T | C | T | A | A | G | G | G | A | C | C | C | C | G | A | T | G | A |  |  |  |  |  |  |  |  |  | C | A | C | G | A | A | C | A |
| #37 | 1 | C | C | C | T | C | T | C | T | A | A | G | A | C | A | C | G | C | C | G | A | T | G | A |  |  |  |  |  |  |  |  |  | C | A | C | G | G | A | T | A |
| #38 | 1 | C | C | C | T | C | T | C | T | A | A | G | G | G | A | C | G | C | C | G | A | T | T | A |  |  |  |  |  |  |  |  |  | C | A | C | G | A | A | C | A |
| #39 | 15 | C | C | C | T | C | T | C | T | A | A | G | G | G | A | C | G | C | C | G | A | T | G | C |  |  |  |  |  |  |  |  |  | C | A | C | G | A | A | C | A |
| #40 | 2 | C | C | T | T | C | T | C | T | A | A | G | G | G | A | C | G | C | C | G | A | T | G | C |  |  |  |  |  |  |  |  |  | C | A | C | G | A | A | C | A |
| #41 | 1 | C | C | T | T | C | A | C | T | A | A | G | G | G | A | C | G | C | C | G | A | T | G | A |  |  |  |  |  |  |  |  |  | C | A | C | G | G | A | T | A |
| #42 | 10 | C | C | C | T | C | T | C | T | A | A | G | G | G | A | C | G | C | C | G | A | T | G | A |  |  |  |  |  |  |  |  |  | A | A | C | G | A | A | T | A |
| #43 | 1 | C | C | C | T | C | T | T | T | A | A | G | G | G | A | C | G | C | C | G | A | T | G | A |  |  |  |  |  |  |  |  |  | A | A | C | G | A | A | T | A |
| #44 | 1 | C | C | C | T | C | T | C | T | A | A | G | G | G | A | C | G | C | C | G | A | T | G | C |  |  |  |  |  |  |  |  |  | C | A | C | G | G | A | T | A |
