# Supplementary material for: Spatial Variation in Genetic Diversity and Natural Selection on the Thrombospondin-Related Adhesive Protein Locus of Plasmodium vivax (PvTRAP)
Source: PLoS One. 2014 Oct 21;9(10):e110463. doi: 10.1371/journal.pone.0110463 (PMC4204863; doi:10.1371/journal.pone.0110463)
Supplement: Table S2 — Characteristics of amino acid substitutions in PvTRAP of worldwide and Thai isolates. (DOC) [file pone.0110463.s002.doc]

**Table S2** Characteristics of amino acid substitutions in PvTRAP of worldwide and Thai isolates

| Amino  acid  position |  |  |  |  |  |  | 1 | 1 | 1 | 1 | 1 | 1 | 1 | 1 | 1 | 1 | 1 | 1 | 1 | 1 | 1 | 1 | 2 | 2 | 2 | 2 | 2 | 2 | 2 | 2 | 3 | 3 | 3 | 3 | 3 | 3 | 3 | 3 | 3 | 3 | 3 | 4 | 4 | 4 | 4 | 4 | 4 | 4 | 4 | 4 | 5 | 5 | 5 | 5 | 5 | 5 | 5 | 5 |
| --- | --- | --- | --- | --- | --- | --- | --- | --- | --- | --- | --- | --- | --- | --- | --- | --- | --- | --- | --- | --- | --- | --- | --- | --- | --- | --- | --- | --- | --- | --- | --- | --- | --- | --- | --- | --- | --- | --- | --- | --- | --- | --- | --- | --- | --- | --- | --- | --- | --- | --- | --- | --- | --- | --- | --- | --- | --- | --- |
|  | 1 | 4 | 7 | 8 | 8 | 8 | 2 | 2 | 2 | 2 | 3 | 3 | 3 | 3 | 4 | 6 | 6 | 7 | 7 | 8 | 8 | 8 | 0 | 0 | 2 | 2 | 5 | 5 | 6 | 9 | 0 | 1 | 2 | 3 | 3 | 4 | 5 | 7 | 7 | 8 | 9 | 0 | 1 | 2 | 2 | 3 | 4 | 4 | 6 | 9 | 1 | 1 | 2 | 3 | 4 | 4 | 5 | 5 |
|  | 5 | 5 | 4 | 2 | 3 | 6 | 0 | 2 | 6 | 9 | 3 | 4 | 5 | 7 | 3 | 0 | 6 | 2 | 6 | 0 | 4 | 6 | 5 | 6 | 3 | 4 | 5 | 8 | 8 | 7 | 3 | 8 | 4 | 5 | 6 | 0 | 2 | 6 | 8 | 0 | 0 | 1 | 3 | 0 | 4 | 8 | 2 | 3 | 7 | 7 | 1 | 8 | 1 | 5 | 7 | 9 | 0 | 1 |
| Salvador I | L | L | **S** | I | **N** | M | **T** | **T** | **T** | M | L | D | E | **Q** | R | V | R | **N** | **Q** | **S** | I | V | E | P | I | K | R | **H** | K | **T** | **N** | K | D | E | G | **N** | A | P | D | **Q** | E | D | **N** | K | **N** | **T** | G | R | E | I | V | V | G | E | A | **Q** | F | K |
| Variants | F | I | G | V | D | K | **S** | **S** | **S** | L | **Q** | E | D | **H** | **Q** | I | **T** | K | K | **T** | V | I | D | L | V | **Q** | K | **Q** | G | A | **S** | **Q** | E | K | R | **T** | D | **S** | G | R | G | **S** | K | R | **T** | A | D | G | A | F | I | I | R | K | V | V | **S** | **S** |
|  |  |  |  |  |  |  |  |  |  |  |  |  |  | R |  |  |  |  |  | **N** |  |  |  | **T** |  |  |  |  |  |  |  |  |  |  | **S** |  |  |  |  |  |  |  |  |  | K |  |  |  |  |  |  |  |  |  |  |  |  |  |
|  |  |  |  |  |  |  |  |  |  |  |  |  |  |  |  |  |  |  |  |  |  |  |  | **S** |  |  |  |  |  |  |  |  |  |  |  |  |  |  |  |  |  |  |  |  |  |  |  |  |  |  |  |  |  |  |  |  |  |  |
|  |  |  |  |  |  |  |  |  |  |  |  |  |  |  |  |  |  |  |  |  |  |  |  | R |  |  |  |  |  |  |  |  |  |  |  |  |  |  |  |  |  |  |  |  |  |  |  |  |  |  |  |  |  |  |  |  |  |  |
|  |  |  |  |  |  |  |  |  |  |  |  |  |  |  |  |  |  |  |  |  |  |  |  | L |  |  |  |  |  |  |  |  |  |  |  |  |  |  |  |  |  |  |  |  |  |  |  |  |  |  |  |  |  |  |  |  |  |  |
| Salvador I | **L** | **L** | **S** | **I** | **N** | **M** | **T** | **T** | **T** | **M** | **L** | **D** | **E** | **Q** | **R** | **V** | **R** | **N** | **Q** | **S** | **I** | **V** | **E** | **P** | **I** | **K** | **R** | **H** | **K** | **T** | **N** | **K** | **D** | **E** | **G** | **N** | **A** | **P** | **D** | **Q** | **E** | **D** | **N** | **K** | **N** | **T** | **G** | **R** | **E** | **I** | **V** | **V** | **G** | **E** | **A** | **Q** | **F** | **K** |
| Variants | **F** | **I** | **G** | **V** | **D** | **K** | **S** | **S** | **S** | **L** | **Q** | **E** | **D** | **H** | **Q** | **I** | **T** | **K** | **K** | **T** | **V** | **I** | **D** | **L** | **V** | **Q** | **K** | **Q** | **G** | **A** | **S** | **Q** | **E** | **K** | **R** | **T** | **D** | **S** | **G** | **R** | **G** | **S** | **K** | **R** | **T** | **A** | **D** | **G** | **A** | **F** | **I** | **I** | **R** | **K** | **V** | **V** | **S** | **S** |
|  |  |  |  |  |  |  |  |  |  |  |  |  |  | **R** |  |  |  |  |  | **N** |  |  |  | **T** |  |  |  |  |  |  |  |  |  |  | **S** |  |  |  |  |  |  |  |  |  | **K** |  |  |  |  |  |  |  |  |  |  |  |  |  |
|  |  |  |  |  |  |  |  |  |  |  |  |  |  |  |  |  |  |  |  |  |  |  |  | **S** |  |  |  |  |  |  |  |  |  |  |  |  |  |  |  |  |  |  |  |  |  |  |  |  |  |  |  |  |  |  |  |  |  |  |
|  |  |  |  |  |  |  |  |  |  |  |  |  |  |  |  |  |  |  |  |  |  |  |  | **R** |  |  |  |  |  |  |  |  |  |  |  |  |  |  |  |  |  |  |  |  |  |  |  |  |  |  |  |  |  |  |  |  |  |  |
|  |  |  |  |  |  |  |  |  |  |  |  |  |  |  |  |  |  |  |  |  |  |  |  | **L** |  |  |  |  |  |  |  |  |  |  |  |  |  |  |  |  |  |  |  |  |  |  |  |  |  |  |  |  |  |  |  |  |  |  |

Amino acid substitutions among Thai isolates are highlighted in light green.

Amino acid property is indicated by colors: red, polar; black, nonpolar; blue, positive charge; pink, negative charge; and brown, neutral.
