# Supplementary material for: Spatial Variation in Genetic Diversity and Natural Selection on the Thrombospondin-Related Adhesive Protein Locus of Plasmodium vivax (PvTRAP)
Source: PLoS One. 2014 Oct 21;9(10):e110463. doi: 10.1371/journal.pone.0110463 (PMC4204863; doi:10.1371/journal.pone.0110463)
Supplement: Table S3 — Minimum number of recombination events (Rm) and recombination sites in the PvTRAP gene of each parasite population in Thailand. (DOC) [file pone.0110463.s003.doc]

**Table S3** Minimum number of recombination events (Rm) and recombination sites in the PvTRAP gene of each parasite population in Thailand

| Province | Rm | Recombination between sites | Spanning domain |
| --- | --- | --- | --- |
| Tak | 3 | 402 and 764 | 2 – 3 |
|  |  | 764 and 1239 | 4 |
|  |  | 1239 and 1325 | 4 |
| Prachuap Khirikhan | 5 | 516 and 764 | 2 |
|  |  | 764 and 1239 | 3 – 4 |
|  |  | 1239 and 1271 | 4 |
|  |  | 1271 and 1325 | 4 |
|  |  | 1325 and 1400 | 4 |
| Chanthaburi | 2 | 516 and 1325 | 2 – 4 |
|  |  | 1327 and 1566 | 4 – 5 |
| Yala and Narathiwat | 2 | 90 and 1019 | 2 – 4 |
|  |  | 1019 and 1327 | 4 |
